# Prolonged use of a proton pump inhibitor reduces microbial diversity: implications for *Clostridium difficile* susceptibility

**DOI:** 10.1186/2049-2618-2-42

**Published:** 2014-11-25

**Authors:** Charlie T Seto, Patricio Jeraldo, Robert Orenstein, Nicholas Chia, John K DiBaise

**Affiliations:** 1 Biomedical Informatics and Computational Biology, University of Minnesota-Rochester, Rochester, MN, USA; 2 Center for Individualized Medicine, Mayo Clinic, Rochester, MN, USA; 3 Institute for Genomic Biology, University of Illinois Urbana-Champaign, Urbana, IL, USA; 4 Division of Infectious Diseases, Mayo Clinic, Scottsdale, AZ, USA; 5 Department of Physiology and Biomedical Engineering, Mayo Clinic, Rochester, MN, USA; 6 Division of Surgery Research, Mayo Clinic, Rochester, MN, USA; 7 Department of Health Sciences Research, Mayo Clinic, Rochester, MN, USA; 8 Division of Gastroenterology, Mayo Clinic, Scottsdale, AZ, USA

**Keywords:** Proton pump inhibitor, Gut microbiome, *Clostridium difficile*

## Abstract

**Background:**

The role of the gut microbiome in arresting pathogen colonization and growth is important for protection against *Clostridium difficile* infection (CDI). Observational studies associate proton pump inhibitor (PPI) use and CDI incidence. We hypothesized that PPI use affected the distal gut microbiome over time, an effect that would be best explored by time-longitudinal study of healthy subjects on PPI in comparison to treatment-naïve CDI subjects. This study enrolled nine healthy human subjects and five subjects with treatment-naïve CDI. After random assignment to a low (20 mg/day) or high (2× 20 mg/day) dose group, fecal samples were collected from the nine healthy subjects before, during, and after 28 days of PPI use. This was done in conjunction with pre-treatment fecal collection from CDI subjects. High-throughput sequencing (16S rRNA) was performed on time-longitudinal samples to assess changes to the healthy gut microbiome associated with prolonged PPI usage. The healthy samples were then compared to the CDI subjects to explore changes over time to the gut microbiome associated with PPI use and potentially related to CDI.

**Results:**

We report that PPI usage at low and high dosages, administered for 28 days, resulted in decreases to observed operational taxonomic unit (OTU) counts after both 1 week and 1 month. This decrease resulted in observed OTU levels that were similar to those found in treatment-naïve CDI patients, which was partly reversible after a 1 month recovery period. We did not detect a dose-dependent difference in OTU levels nor did we detect significant changes in taxa previously reported to be affected by PPI treatment.

**Conclusion:**

While our observation of diminishing observed OTU counts during PPI therapy is a preliminary finding in a small cohort, our hypothesis that PPIs disrupt the healthy human gut microbiome is supported in this group. We conclude that decreases in observed species counts were reversible after cessation of PPI usage within 1 month. This finding may be a potential explanation for the association between prolonged PPI usage and CDI incidence.

## Background

Proton pump inhibitors (PPIs) are potent inhibitors of gastric acid production that are highly effective for treating acid-mediated disorders of the upper digestive tract [1]. PPIs are one of the most commonly prescribed medications in the USA, with billions of dollars in annual sales [2]. Although they have a long history of safety and efficacy [3], epidemiological studies have linked PPIs to several nutritional, metabolic, and infectious disorders. Specifically, their prolonged use has been associated with iron [4] and vitamin B12 [5,6] deficiencies, hypomagnesemia [7], osteoporosis-related fractures [8,9], small intestinal bacterial overgrowth [10,11], and community-acquired pneumonia [12,13]. Furthermore, several recent studies have found an association between the use of PPIs and the development of *Clostridium difficile* infection (CDI) [14-18]. A meta-analysis of 39 studies reported a 1.74-fold increase in risk of CDI in PPI users (95% confidence interval 1.47-2-05) [19]. This association with the most common healthcare-acquired infection led the U.S. Food and Drug Administration (FDA) to require that the package insert for PPIs contain a warning that PPIs may increase the risk of CDI [20].

Despite this epidemiological association, the reason why PPIs might increase CDI risk is not known. Although hypochlorhydria increases susceptibility to enteric bacterial infections, *C. difficile* spores are unaffected by gastric acidity [21]. The intestinal microbiota play an important function in suppressing pathogen growth (i.e., colonization resistance) [22], and alterations in the microbiota by PPIs may induce *C. difficile* proliferation. The loss of colonic microbial diversity is a characteristic feature of CDI [23,24]. Microbiome analysis of the feces has been shown to improve the ability to distinguish CDI status when comparing cases with both diarrheal and non-diarrheal controls [25]. Prolonged use of PPIs has been linked to changes in the microbial community composition of the upper intestinal tract *in vitro*[10,11,26,27]. Gastric acid reduction may also influence the microbial composition of the lower gastrointestinal (GI) tract [26]. The longitudinal changes in the gut microbial ecology during and after PPI use and how these relate to the microbiota present in CDI remain unknown.

The purpose of this study was to investigate the impact of short- and long-term PPI use on the fecal microbiota and to compare these changes to persons with newly diagnosed CDI. If similar, it would provide a potential mechanism for the association of PPIs with an increased risk of CDI. Changes to the fecal microbial community induced by PPI use were assessed in healthy humans using high-throughput 16S hypervariable tag sequencing and compared to patients with a treatment-naïve first episode of CDI. Our results demonstrate that microbial diversity declines following prolonged (28-day) PPI usage. This decrease results in observed operational taxonomic unit (OTU) levels that are similar to those found in treatment-naïve CDI patients. The reduction in observed OTU levels was not always reversible 1 month after ceasing treatment.

## Results

### Study participants

Ten healthy volunteers (five women, five men) were randomly assigned to receive omeprazole 20 mg once daily (*n* =5) or twice daily (*n* =5) for 28 days. Volunteers ranged in age from 18 to 57 years (mean ± standard deviation: 40.8 ± 15.3 years), with body mass indices (BMI) ranging from 18.6 to 29.7 kg/m^2^ (mean ± standard deviation: 24.8 ± 3.7 kg/m^2^). Stool samples were collected prior to baseline (T0) and following 7 days (T7) and 28 days (T28) of omeprazole use. A final stool sample was collected 1 month after discontinuation of omeprazole (T56). Fecal DNA was sequenced and compared to that from five treatment-naïve CDI patients, all women, ranging in age from 20 to 63 years (mean ± standard deviation: 40.8 ± 15.9 years) with BMI ranging from 16.6 to 39.4 kg/m^2^ (mean ± standard deviation: 28.1 ± 8.7 kg/m^2^).

No adverse events were reported amongst the healthy subjects receiving omeprazole. One healthy subject was withdrawn from the study after providing the baseline and the first on-PPI stool samples due to subsequent antibiotic use prescribed by his primary care provider.

The OTUs corresponding to *Clostridium* in CDI subjects were assessed to determine if *C. difficile* could be detected across all five subjects. *C. difficile BI1* was detected in 4/5 CDI subjects (as OTU 57). Other major *Clostridium* associated with CDI subjects included *Clostridium butyricum* (as OTU 377) in 4/5 CDI subjects and *Clostridium perfringens* (as OTU 393) in 3/5 CDI subjects.

### Sequencing

The total number of utilized read-pairs per sample from healthy volunteers and CDI patients was 14,635,736 (mean ± standard deviation 340,366 ± 500,176). The lowest read-count of a sample was 75,506 (see Additional file 1: Metadata). Rarefaction plots measuring observed-OTU diversity indicate that all samples were sequenced past exponential species accumulation (Additional file 2: Figure S1).

### PPI use is associated with reduced OTU diversity

**Figure 1:**
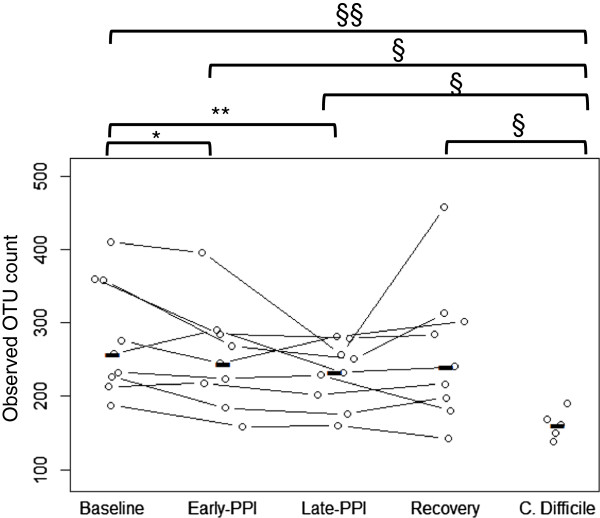
**Observed OTU counts on proton pump inhibitors.** Plot depicts patient observed-OTU counts at start of treatment, 1 week into treatment, and at the end of the 1-month program, followed by final collection 1 month after ceasing PPI usage. Five first-incidence *C. difficile* patients had stool isolated and sequenced to represent the *C. difficile* cohort. *Black horizontal bars* represent within-group means. All statistical tests using matched-pair healthy volunteers used Wilcoxon signed rank; statistical tests between healthy volunteer groups (*p <0.05, **p <0.005) and *C. difficile* group used Wilcoxon rank sum (§*p* <0.05, §§*p* <0.01).

After rarefaction of all samples to 75,000 reads, a significant difference in observed OTU count between baseline and 1 month was observed (Figure 1, Table 1). To determine the robustness of this observed difference to sampling depth, sensitivity analysis was performed by generating additional rarefactions of 50,000 and 25,000 reads/sample to assess the effect of reduced read depth (Additional file 2: Figure S2). The significant change in observed species count between baseline and 1 month on PPI remained significant at 50,000 and 25,000 reads/sample (Additional file 2: Table S1).

**Table 1:** Statistical significance of observed OTU changes between sample groups

**Cohort**	**T0**	**T7**	**T28**	**T56**
T0				
T7	0.0117			
T28	0.0039	0.0328		
T56	0.0328	0.9102	0.1069	
CD^a^	0.0051	0.0112	0.0829	0.0599

Table of statistical tests between matched-pair healthy subject time intervals to determine the significance of measured differences in observed species counts. Species counts were calculated from samples rarefied to 75,000 reads. Testing for matched-pair healthy samples used one-tailed (alternative = “greater”) Wilcoxon signed rank.

^a^Non-matched pair probabilities for groups with *C. difficile* (CD) were calculated using one-tailed (alternative = “greater”) Wilcoxon rank sum with continuity correction.

### Microbial diversity differences between genders in PPI users

After dividing subjects by gender and comparing observed OTU counts across all time points, a statistically significant difference between observed OTU counts in men (*n* =4) and women (*n* =5) on PPIs was found (*p* ≈ 0.004, Additional file 2: Figure S3). However, when observed OTU counts at each time point (T0, T7, T28, T56) for men and women were compared, the differences observed were not significant (*p* ≈ 0.10, *p* ≈ 0.21, *p* ≈ 0.10, *p* ≈ 0.10). For observed OTU counts, the mean ± standard deviation for women was 226 ± 55 and 293 ± 77 for men.

### No PPI dose-dependent effect on diversity

A comparison of OTU counts between the high (twice daily PPI) and low (once daily PPI) dose groups revealed no significant difference in observed OTU counts (i.e., prior to omeprazole use) between the groups at baseline, 7 days on PPI, 28 days on PPI, or 1 month after stopping the PPI (*p* ≈ 0.2778, *p* ≈ 0.2063, *p* ≈ 0.5556, *p* ≈ 0.2778, respectively). After separating subject time points into two groups based on dosage, no significant difference was detected between the 20-mg once/day and 20-mg twice/day groups (*p* ≈ 0.072); mean ± standard deviation for the once daily omeprazole group was 239 ± 62 and for the twice daily omeprazole group was 277 ± 81 (Additional file 2: Figure S4).

### Longitudinal gut microbe composition is largely stable

After rarefaction of all samples to 75,000 reads, the percent of OTUs that remained throughout the course of the PPI therapy varied between 14.33% and 37.38% (median ± MAD of 25.3% ±9.5%) across the nine subjects, corresponding to longitudinal trend 1111 (Additional file 2: Table S2). Of the total OTUs observed in the subjects, 4.08%–14.13% (Median ± MAD of 7.7% ±1.6%) appeared after the conclusion of the PPI therapy, corresponding to longitudinal trend 0001.

### Similarity of OTU richness between PPI use and CDI

Using subject data rarefied to 75,000 reads/sample, observed OTU diversity was compared between the five CDI subjects as well as the time points of male or female subjects (Additional file 2: Table S3). The change in observed OTUs in CDI subjects and all healthy subjects were also compared by time interval (Table 1). We reported marked differences in observed OTU counts between healthy subjects at baseline and those with CDI that erode after 1 week and 1 month of PPI therapy (Table 1). This difference in observed OTU counts is also apparent when the healthy subjects are subdivided into male and female groups (Additional file 2: Table S3).

### No compositional overlap between PPI use and CDI

**Figure 2:**
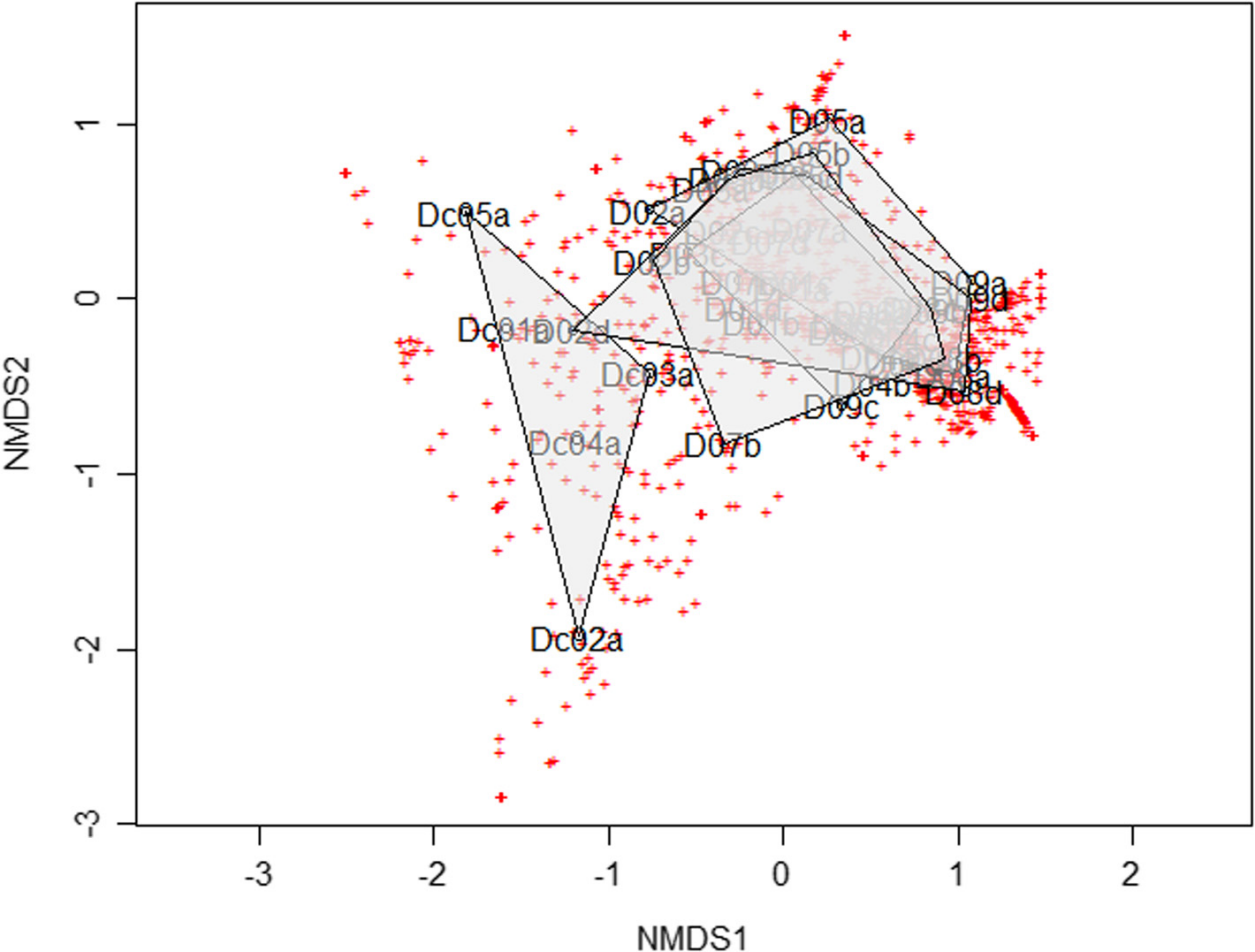
**No overlap of on-PPI and CDI subjects.** NMDS plots show no taxonomic overlap between short- and long-term PPI users and subjects with *C. difficile* infection, with strong overlap between all subjects and all time points.

Non-metric multidimensional scaling (NMDS) and SourceTracker were used to determine if healthy subjects using a PPI retained microbial ecological similarity to baseline samples or if a CDI-like microbial composition could be detected. Compositional similarity to the CDI patients was potentially indicated in one of the subjects (D02d, Figure 2). The NMDS plots showed strong overlap of on-PPI time points (Figure 2). A SourceTracker calculation indicated the closest sink to on-PPI or after-PPI samples was baseline compared to the five CDI subjects, and an “Unknown” group as visualized in a ternary plot (Additional file 2: Figure S5).

### No detectable PPI-induced pathway changes

**Figure 3:**
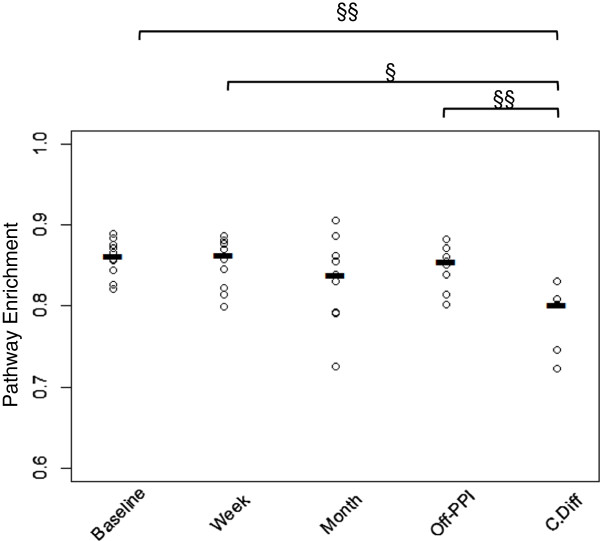
**KEGG Pathway disruptions on PPI.** Plot depicts relative enrichment of KEGG pathway “Glycine, Serine, Threonine Metabolism” at start of treatment, one week into treatment, and at the end of the one-month program. The difference in relative abundance of the pathway for the duration of treatment did not vary significantly, with significant difference in means between subjects with *C. difficile* and other time points except the one-month group (§ *p* <0.05; §§ *p* <0.005).

The nearest sequenced taxon index (NSTI), a metric to determine divergence of taxonomic reads from closed references, was calculated per sample with mean ± standard deviation of 0.07 ± 0.03. These reported values are within the range of the NSTI generated from the phylogenetic investigation of communities by reconstruction of unobserved states (PICRUSt) benchmarking of mammalian gut samples (0.14 ± 0.06) [28]. No significant change in enrichment in any of the Kyoto Encyclopedia of Genes and Genomes (KEGG) pathways was detected during PPI treatment of healthy subjects. However, there was a significant enrichment in glycine-serine-threonine metabolism in healthy patients pre-PPI use compared to the CDI subjects (*p* ≈ 0.001, Figure 3, Additional file 2: Table S4). The enrichment of this pathway decreased during the course of PPI treatment until it was not significantly different from the CDI group, and the enrichment of this pathway increased during recovery until significantly it was different from the CDI subjects (*p* ≈ 0.007, Figure 3, Additional file 2: Table S4).

### Individual ecological distinctiveness is preserved

**Figure 4:**
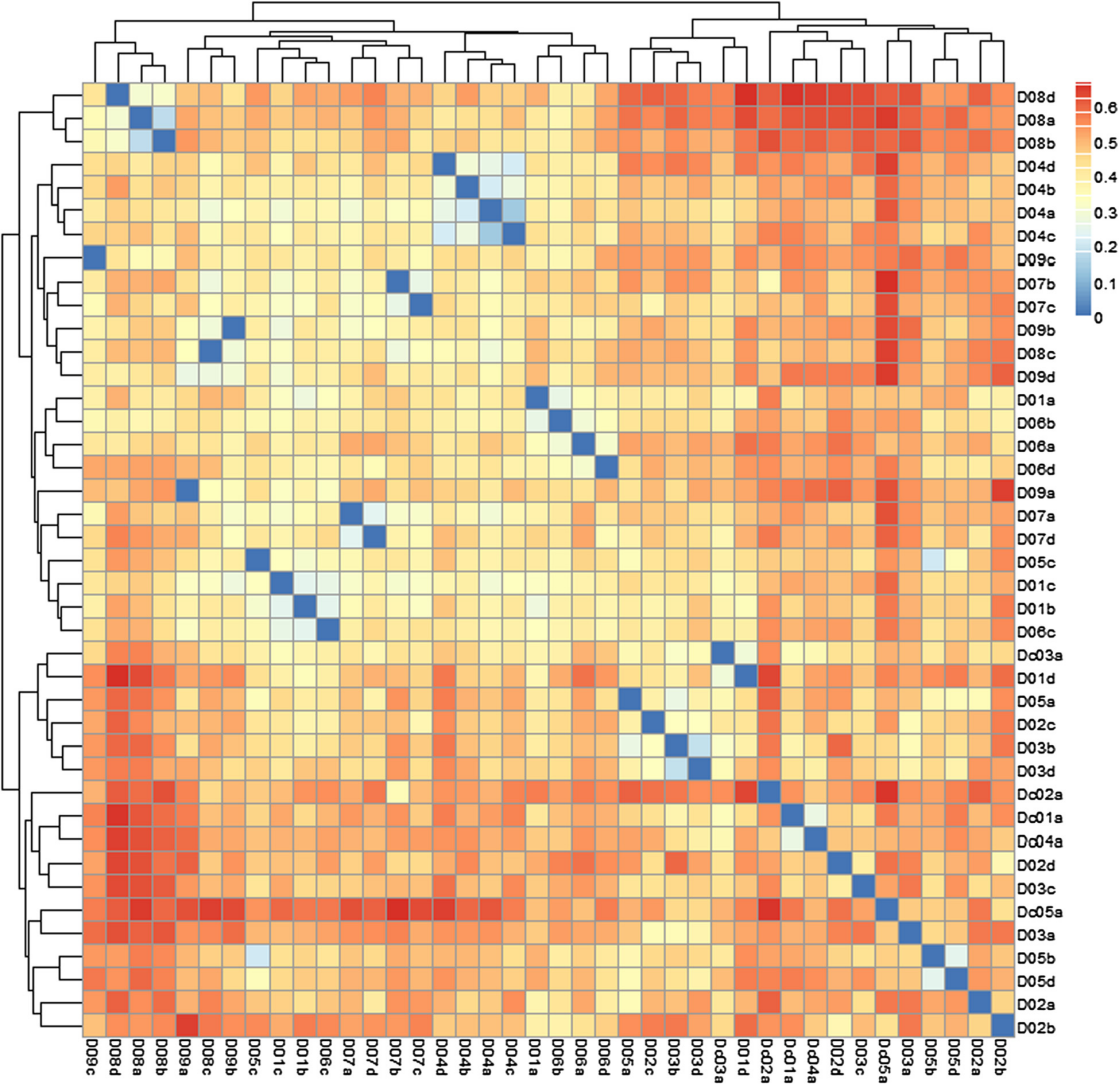
**Beta diversity of samples.** Beta diversity *via* unweighted UniFrac of samples from healthy volunteers and *C. difficile* patients was calculated for all samples against all samples to generate a distance matrix. Samples were then clustered by Euclidean distance. CDI patients (4/5) formed a cluster distinct from the healthy volunteers and cluster by patient more than time point.

The unweighted UniFrac β-diversity distance matrix was calculated from a sample rarefied to 75,000 reads/sample (Figure 4), with one subject samples remaining most similar to each other for the duration of treatment (subjects 4). Three subjects had their 1-month sample as the outlier (subjects 6, 8). Four CDI subjects (*C. difficile* (CD) subjects 1, 2, 4) clustered closely to each other, with CDI subject 3 forming a sub-cluster with healthy subject 1’s post-treatment sample (D01d) and CDI subject 5 clustering with subject 3 at baseline (D03a).

## Discussion

We conducted a study that longitudinally tracked the fecal microbiome of healthy humans before, during, and 1 month after a once- or twice-daily PPI use to investigate the hypothesis that PPI use can cause perturbations to the healthy gut microbiome that bear similarity to the microbial changes that occur in CDI. While limited by a small sample size, we observed that PPIs induce a marked and persistent decrease in observed OTU count, a surrogate for microbial diversity. At the end of the 28-day treatment period, the observed OTU counts were similar to those from patients with a first CDI episode indicating the potential for PPIs to negatively influence the robustness of the host microbial ecology and increase the susceptibility to CDI.

This is the first study to examine the impact of PPI use on the human gut microbiota using high-throughput sequencing. Our findings are consistent with past longitudinal studies of the GI microbiome that indicate stable individual microbial communities [29]. That is to say, despite the effects of PPI use, samples from the same subject taken over time remained more similar to each other than to the samples obtained from the other healthy subjects. In our work, this is supported by the unweighted UniFrac β-diversity distance matrix (Figure 4) showing clusters comprised of individual samples. NMDS plots also show minimal movement of individual samples over time, despite the overall reduction in diversity over time with PPI use.

Garcia-Mazcorro et al. showed that PPI usage in canines resulted in an increase of *Lactobacillus* spp. in male (*n* =4) and female dogs (*n* =4) and a decrease in *Faecalibacterium* spp. and *Bacteroides-Prevotella-Porphyromonas* in the male dogs only [30]. In contrast, our comparison of taxa highlighted in Garcia-Mazcorro et al. revealed no significant differences during PPI treatment (Additional file 2: Table S5). The lack of taxa-specific associations in our human cohort may be due to the larger baseline differences that arise due to differences in age, environment, diet, and starting microbiomes, effects that are more difficult to overcome with small sample sizes. This variability can be observed in changes of relative abundance of taxa over time that varies between subjects (Additional file 2: Figure S6). Garcia-Mazcorro et al. also collected mucosal biopsy samples from the stomach and duodenum of the dogs, reporting a decrease in *Helicobacter* spp. and a corresponding increase in the relative abundance of other bacteria in the stomach. However, in the duodenum, an overall increase in the abundance of all bacteria (i.e., small bowel bacterial overgrowth) was seen. Garcia-Mazcorro et al. did not report major qualitative changes in the phylogenetic composition of stomach and duodenal microbial communities after PPI use. A limitation of our study is that, given the complexities and expense of obtaining specimens from the upper GI tract, we did not evaluate the upper gut microbiota, an area of particular interest given the proximity to the acid-producing stomach. Further study in this area is needed.

Kanno et al. administered omeprazole to mice and found dose-dependent changes in fecal microbial communities [26]. In contrast to the study of Garcia-Mazcorro, they found a higher abundance of all groups of fecal bacteria with the exception of *Bifidobacterium* during omeprazole use. Because it is unlikely that omeprazole reaches the colon intact, it was speculated that the microbial changes observed in the feces relate to an increase in bacterial load entering the colon or to changes in dietary protein reaching the colon given the role of gastric acid in the early stage of protein digestion.

Direct effects of PPIs on gut microbes may provide an explanation for the observed changes to community structure and species diversity during treatment, and may also provide a potential mechanism whereby PPIs increase the risk of CDI. Bacterial counts of many species increase in the setting of gastric hypochlorhydria. In contrast, omeprazole can inhibit the growth of many gut microbial species [31]. Loss of microbial diversity is a consistent feature in CDI patients [32] and animal models of antibiotic-mediated CDI [33]. *In vivo* antibiotic treatment leads to an increase in gut sialic acid, a favored catabolite of *C. difficile* whose availability is strongly associated with *C. difficile* bacterial load [34]. The published literature provides strong evidence for the role of nutrient competition between the gut microbiome and *C. difficile* in utilization of available amino acids [35], monomeric glucose, *N*-acetylglucosamine, and sialic acids [36]. Although disruption of a healthy gut microbiome can create an environment conducive to *C. difficile* proliferation, the specific changes in host and microbial factors that directly lead to *C. difficile* invasion in humans remain unclear.

In addition to eliminating nutrient competitors, PPI use may directly affect gene expression across metabolic pathways in favor of *C. difficile* growth. We detected a significant decrease in enrichment of the KEGG pathway “glycine-serine-threonine metabolism” in CDI subjects compared to the healthy subjects. This pathway combines the synthesis of threonine and serine from separate pathways and the final production of glycine from either amino acid [37]. Glycine is a well-known enhancer of *C. difficile* spore germination [38]. Glycine and threonine supplementation has been shown to increase *in vitro* bacterial load and together lead to markedly increased *C. difficile* toxin production [39]. Given these effects, the reduction of enrichment of glycine-serine-threonine in CDI is unexpected, and future work may attempt to directly quantitate amino acid concentrations in the gut in CDI subjects and GI-healthy subjects. The lack of significant changes in enrichment of this pathway in healthy subjects is expected given the lack of adverse effects reported by the healthy subjects. Further study is warranted to investigate the premise that PPI use can alter production of metabolites that serve as *C. difficile* germinants, growth factors, and toxin production enhancers.

Specific bile salts and glycine act as co-germinants of *C. difficile* spores, converting them to vegetative (infectious) forms. *In vivo* and independent of *C. difficile*, glycine appears to alter the ratio of deconjugated (primary bile salts) to conjugated (secondary bile salts) in the intestine [22]. In contrast, secondary bile salts such as deoxycholate prevent vegetative growth [38,40]. After severe disruption of gut microbial populations by PPI-induced hypochlorhydria, secondary bile salts are significantly reduced, allowing unrestricted vegetative growth [41]. Chenodeoxycholate, a primary bile salt, inhibits germination and protects the small intestine by inhibiting production of vegetative forms, until spores reach the anaerobic environment of the large bowel [42]. These conditions are reversed by cholestyramine, which binds bile salts that stimulate spore germination [43].

The direct effect of PPIs on *C. difficile* can be divided into effects on spore germination, effects on growth, and effects on toxin production. A recent report found that *C. difficile* spore germination in gastric contents was increased by nutrient supplementation as opposed to PPI use but did not compare germination efficacy with and without PPI [44]. No research is currently available to describe the effect of PPI on *C. difficile* spore germination or vegetative growth, but PPIs have a notable effect on toxin production of vegetative *C. difficile. In vitro*, some strains of *C. difficile* possess enhanced toxin production in the presence of omeprazole, independent of final pH [45]. Strain-level variations in enhanced toxin production of omeprazole are likely to be an explanation of why PPI use is not as strongly associated with CDI as other well-known risk factors.

## Conclusions

In conclusion, we have shown that PPI use in humans reduces microbial diversity, a condition found in subjects with CDI. A follow-up study with a greater number of recruited subjects will improve our confidence in this finding, a necessary precursor to future studies. Further investigation into the role of PPIs in altering metabolism in gut bacteria is likely to solidify our understanding of PPI-mediated changes within the gut microbiome and provide additional insight into the association between prolonged PPI usage and CDI acquisition. Finally, additional research should be performed in an effort to mitigate the loss of microbial diversity consequent to PPI use.

## Methods

### Ethics statement

The study was approved by the Mayo Clinic Institutional Review Board (#13-000180) and registered on ClinicalTrials.gov (NCT01822977). Written informed consent was provided by all individuals enrolled.

### Study participants

Study participants were recruited at Mayo Clinic in Arizona. Ten volunteers without acute or chronic GI symptoms or conditions and five adult patients experiencing their first episode of CDI were enrolled. Healthy subjects were screened for current or chronic GI symptoms using a 16-item questionnaire. Only those with an absence of symptoms were eligible to participate. Exclusion criteria for healthy volunteers included prior surgery altering the esophagus, stomach, and intestine; chronic daily use of medications affecting GI secretion or motor function; the presence of any GI-motility affecting systemic diseases or untreated psychiatric disease; and pregnancy. Use of the following medications was prohibited: antibiotics within 2 months of the start of the study, probiotics within 2 weeks, and chronic use of medications that alter gastric acid (PPI, histamine-2-receptor antagonists) or motility (prokinetic agents, narcotic analgesics, laxatives, anticholinergics, antidiarrheals). Those on a stable dose of daily fiber were allowed to participate. In the *C. difficile* group, only patients experiencing their first episode of CDI who had not yet been treated were eligible to participate.

### Study design

Following consent, a fresh stool sample was collected from the ten healthy subjects (T0). These ten subjects were then randomly assigned to receive omeprazole 20 mg once daily (*n* =5) or twice daily (*n* =5) for 28 days. Stool samples were collected after 7 (T7) and 28 (T28) days of omeprazole use. A final stool sample was collected 1 month after discontinuation of omeprazole (T56). Additionally, fresh stool samples were collected from five treatment-naïve CDI subjects before commencing treatment.

### Sample collection and storage

Subject stool samples were passed into a stool collection device at the subject’s home in the morning, with the subject transferring a portion into a specimen container. The specimen container was brought to the Mayo Clinic in Arizona study coordinator fresh within 6 hours of passing. *C. difficile* samples were taken from consented patients clinical specimens after *C. difficile* testing was completed. At the Mayo Clinic in Arizona, the specimen was frozen at −80°C before being shipped to the Mayo Clinic in Rochester in an insulated container with dry ice. At the Mayo Clinic in Rochester, samples were stored at −80°C until DNA extraction.

### DNA extraction and library preparation

DNA was extracted from samples using the MoBio PowerSoil Kit (MoBio Laboratories, Carlsbad, CA, USA). Genomic DNA (gDNA; 50 ng) was used as template for a polymerase chain reaction (PCR) with 0.3 uM V3-V5 barcoded primers [46] targeting 357 F and 926R (5′AATGATACGGCGACCACCGAGATCTACACTATGGTAATTGTCCTACGGGAGGCAGCAG3′ and 5′CAAGCAGAAGACGGCATACGAGATGCCGCATTCGATXXXXXXXXXXXXCCGTCAATTCMTTTRAGT3′, respectively) of the bacterial 16S gene, along with Kapa HiFi Hotstart Ready Mix (Kapa Biosystem). PCR conditions were set to 95°C/3 min, 35 cycles of 98°C/30 s, 70°C/15 s, 72°C/15 s, and finally 72°C/5 min in a Bio-Rad T100 Thermal Cycler. PCR product sizes were verified using Agilent Tapestation with reaction cleanup and DNA was purified using an epMotion automated system (Eppendorf) with Agencourt AMPure PCR Purification System. Final quantitation was performed using QuBit HS dsDNA kit using the QuBit 2.0 flurometer (Life Technologies). Samples were pooled to equal concentrations and sequenced on one lane of MiSeq at the Mayo Genomics Facility using MiSeq Reagent Kit v2 (2 × 250 reads, 500-cycles) (Illumina Inc., San Diego, CA).

### NGS data analysis

Quality control of 16S reads can strongly effect ecological diversity metrics such as alpha- and beta-diversity, with filtering of low quality reads playing an important role in reducing the count of spurious OTU detection [47]. Briefly, after quality-filtering using Trimmomatic, paired 16S reads were de-replicated and singletons and chimeras were removed and clustered by similarity with representative sequence selection with USEARCH, part of the UPARSE package [48]. Benchmarks by the author of the UPARSE package against mock OTU sets suggest that UPARSE better approximates actual species diversity due to its conservative nature. Representative sequences representing the OTUs were classified using Greengenes [49] 13_5 database with mothur 1.32.1 [50]. Clean sequences were re-mapped to the representative OTUs using USEARCH. Output files were converted to BIOM format and processed using QIIME 1.7.0 [51]. An observed species rarefaction curve was generated to measure saturation of sequencing (Additional file 2: Figure S1).

As the default Greengenes database did not possess *C. difficile* representatives, 72 sequences and appropriate taxonomy were downloaded from SILVA SSU r117 [52] by searching the online version of r117 for “clostridium difficile” for non-repetitive reference sequences (Ref(NR)). The sequences and taxonomic data were concatenated to the reference fasta and taxonomy files, respectively. This supplemented Greengenes99 file was then used to re-classify the OTU representative reads from USEARCH in mothur using the classify.seqs function.

The R function sample was used to perform random sampling with replacement. The input used was a vector of incrementing values from 1 to a number equal to the total number of OTUs in the community matrix. For each sample, the probability of selecting any OTU was weighted by the relative abundance of that OTU in a particular sample. OTUs were then randomly selected 75,000 times for each sample, a value less than the read-count of the sample with the lowest read-count (75,506). The list of randomly sampled OTUs per subject was converted into a down-sampled community matrix by taking the randomly sampled OTU counts and incrementing the rows of a blank community matrix corresponding to the randomly selected OTUs.

### Comparison of healthy and CDI datasets

SourceTracker estimates the proportion of a sampled microbial community that is derived from defined source communities. Previous uses include estimating the proportion of the microbiota detected at a variety of “sinks” from various environmental “sources” [53]. SourceTracker 0.95 from Quantitative Insights Into Microbial Ecology (QIIME) was used on unrarefied data to determine the relatedness of various sink samples (T7, T28, T56) to sources (T0, CD). The mapping file previously used with QIIME was appended to include the additional columns required to designate sources and sinks. The healthy subjects and CDI subjects at baseline were used as sources, with healthy subjects at 7-days, 28-days, and 1-month post-treatment treated as sinks. SourceTracker performed rarefaction of 75,000 reads with ten restarts and 100 burn-in iterations. The percent relationship between the aggregate sinks and the aggregate CDI and T0 sources were plotted on a ternary plot constructed using the R package vcd.

NMDS was selected to analyze the between-sample distances of the community matrix [54]. NMDS uses rank-order of pairwise distances in a distance matrix and is a more robust technique for measuring relatedness of samples [55]. NMDS analysis was used to determine variation between CDI- and PPI-consuming subjects over time using a community matrix rarefied to 75,000 reads/sample. The R package vegan was used for NMDS calculations; specifically, function metaMDS was used to perform the NMDS calculation, followed by shape generation with ordihull and labeling of samples via orditorp. Shapes were generated based on sample group and time point, comprising CDI subjects, healthy subjects at baseline (T0), subjects after 1 week of PPI treatment (T7), 1 month of treatment (T28), and 1 month after ceasing PPI treatment (T56).

### Diversity measures

Taking biom and tree files generated from UPARSE and mothur, data were converted to tabular form and analyzed using R-3.1.0 on R-Studio 0.98. Observed species counts were calculated using function “specnumber” from package vegan. Shannon diversity was calculated using function “diversity” with index = “shannon” from package vegan. The rarefaction depth utilized for subsequent statistical analyses was 75,000 reads/sample. Chao diversity was calculated using function specpool with index = “chao” from package vegan. After rarefaction in QIIME, unweighted UniFrac β-diversity was calculated via beta_diversity.py in QIIME. The distance matrix was exported to R and visualized using heatmap, using Euclidean distance to cluster rows and columns.

### Statistical testing

To test for normality of the data, the skewness function from R library e1071 [56] was used to evaluate the distribution of observed OTU data. Non-parametric Wilcoxon tests were chosen to evaluate statistical significance of observed differences between datasets. Between healthy subjects longitudinally paired data, one-tailed Wilcoxon signed rank was used to evaluate if observed OTU diversity decreased during PPI use and was still lower than at baseline. The Wilcoxon rank sum test with continuity correction was used to compare non-matched CDI subjects and healthy subjects at various time points. Wilcoxon rank sum was also used to compare high and low dosage groups and observed OTU counts between men and women.

### Synthetic metagenome prediction

PICRUSt was used to infer metagenomes from the 16S data [28]. From the 16S data we extrapolated gene content based on the evolutionary distance of a detected OTU to the closest relative with a completed reference genome. Very close evolutionary relationships to an organism with a closed reference genome would improve the confidence of predicted genome content, in contrast to OTUs with a closed reference genome only available from a distant evolutionary relative. The distances of all taxa to the nearest organism with closed reference genome were summed to derive the NSTI for each sample. OTU picking was re-performed in QIIME using pick_closed_reference_otus.py. The resulting biom file was then normalized using normalize_otus.py to convert the OTU content into KEGG gene content. Pathway annotation was generated using predict_metagenomes.py with the normalized biom file as input with default output using KEGG pathway information [37]. KEGG pathway data was then exported in tabular form to R-3.1.0 in R-Studio 0.98 for subsequent analysis.

### Longitudinal changes

Evaluation of longitudinal trends was performed by transforming numeric counts per OTU into presence/absence (1/0) for each subject’s four time points (baseline, short-term PPI, long-term PPI, post-PPI recovery). Absence (“0”) was strictly defined as no reads detected, with presence (“1”) defined as detectable reads per OTU for a given subject. For two outcomes and four time points, this resulted in 2^4^ − 1 combinations of an OTU after subtracting the combination corresponding to the absence in all time points (0000). From a rarefied community matrix of 75,000 reads per sample, the behavior of each OTU in each subject was classified into 1 of 15 bins corresponding to combinations of presence and absence at each time point (0001, 0010, 0100, 1000, 1111, etc.). The number of OTUs that were classified as belonging to each bin were calculated and plotted to assess the effects of PPI usage on multiple species per person.

Separately, longitudinal changes to relative abundance after commencement of treatment were calculated by taking the log10 transform of relative abundance per taxa at each post-treatment time point for each individual and dividing by the relative abundance of that taxa at baseline. For transforms that equaled infinity, the value was fixed at +10. For transforms equaling negative infinity, the value was fixed at −10.

## Availability of supporting data

Sequences are available at NCBI SRA (SRA: SRP045730; BioProject: PRNJA259188). The metadata associated with the sequences can be found in the additional files (Additional file 1: Metadata).

## Abbreviations

PPI: proton pump inhibitor; OTU: operational taxonomic unit; CDI: *Clostridium difficile* infection; NSTI: nearest sequenced taxon index; MAD: median absolute deviation.

## Competing interests

The authors declare that they have no competing interests.

## Authors’ contributions

Conceived and designed the experiments: JD, NC, and RO. Performed the experiments: CS. Analyzed the data: CS, NC, and JD. Contributed reagents/materials/analysis tools: PJ. Wrote the paper: CS, JD, RO, and NC. All authors read and approved the final manuscript.

## Supplementary Material

Additional file 1 **Metadata.** Table showing sample metadata, along with the number of paired reads after quality control. Click here for file

Additional file 2 **Supplemental Figures and Supplemental Tables.** Figures include per-sample observed OTU rarefaction plots **(Figure S1)**, rarefaction sensitivity plots **(Figure S2)**, PPI effects on gender **(Figure S3)**, and dosage **(Figure S4)**. SourceTracker ternary plot describes relationship of on-PPI samples to CDI and baseline **(Figure S5)**. Differential change at family level over time per subject presented on heatmap **(Figure S6)**. Time-longitudinal plots for Shannon **(Figure S7)** and Chao **(Figure S8)** diversity relative to CDI. Tables include statistics for rarefaction sensitivity analysis **(Table S1)** and longitudinal OTU bins per subject **(Table S2)**. Table of statistical tests for gender effects of PPI **(Table S3)**, KEGG pathway enrichment **(Table S4)**, changes in enrichment for Garcia-Mazcorro taxa **(Table S5)** and longitudinal changes to Chao and Shannon diversity **(Table S6)**. Click here for file
