# Erratum to: Prolonged use of a proton pump inhibitor reduces microbial diversity: implications for *Clostridium difficile* susceptibility

**DOI:** 10.1186/s40168-016-0158-1

**Published:** 2016-02-22

**Authors:** Charlie T. Seto, Patricio Jeraldo, Robert Orenstein, Nicholas Chia, John K. DiBaise

**Affiliations:** Biomedical Informatics and Computational Biology, University of Minnesota-Rochester, Rochester, MN USA; Center for Individualized Medicine, Mayo Clinic, Rochester, MN USA; Institute for Genomic Biology, University of Illinois Urbana-Champaign, Urbana, IL USA; Division of Infectious Diseases, Mayo Clinic, Scottsdale, AZ USA; Department of Physiology and Biomedical Engineering, Mayo Clinic, Rochester, MN USA; Division of Surgery Research, Mayo Clinic, Rochester, MN USA; Department of Health Sciences Research, Mayo Clinic, Rochester, MN USA; Division of Gastroenterology, Mayo Clinic, Scottsdale, AZ USA

After publication of this article [1], an error was reported in the ‘Methods’ section of the article under the subheading ‘DNA extraction and library preparation’. The second primer associated with 926R was incorrectly stated as: “CAAGCAGAAGACGGCATACGAGATGCCGCATTCGATXXXXXXXXXXXXCCGTCAATTCMTTTRAGT”. The correct sequence for the experimental work was: “CAAGCAGAAGACGGCATACGAGAT NNNNNNNNNNNN AGTCAGTCAG CC CCGTCAATTCMTTTRAGT”.
