# Supplementary material for: Prolonged use of a proton pump inhibitor reduces microbial diversity: implications for Clostridium difficile susceptibility
Source: Microbiome. 2014 Nov 25;2:42. doi: 10.1186/2049-2618-2-42 (PMC4242847; doi:10.1186/2049-2618-2-42)
Supplement: Additional file 2 — Supplemental Figures and Supplemental Tables. Figures include per-sample observed OTU rarefaction plots (Figure S1), rarefaction sensitivity plots (Figure S2), PPI effects on gender (Figure S3), and dosage (Figure S4). SourceTracker ternary plot describes relationship of on-PPI samples to CDI and baseline (Figure S5). Differential change at family level over time per subject presented on heatmap (Figure S6). Time-longitudinal plots for Shannon (Figure S7) and Chao (Figure S8) diversity relative to CDI. Tables include statistics for rarefaction sensitivity analysis (Table S1) and longitudinal OTU bins per subject (Table S2). Table of statistical tests for gender effects of PPI (Table S3), KEGG pathway enrichment (Table S4), changes in enrichment for Garcia-Mazcorro taxa (Table S5) and longitudinal changes to Chao and Shannon diversity (Table S6). [file 2049-2618-2-42-S2.pptx]

## Slide 1
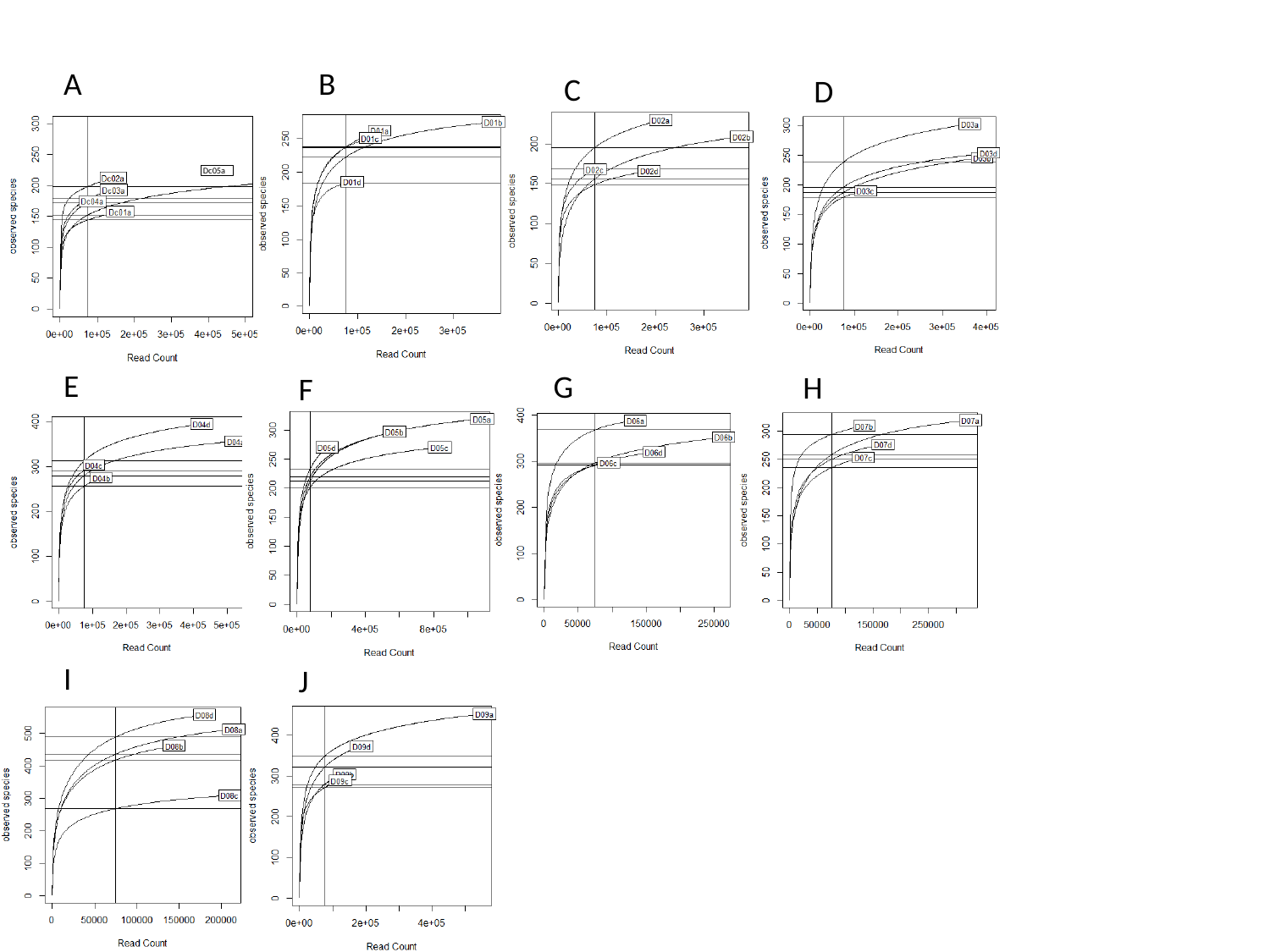

A
B
C
D
Dc05a
E
G
H
F
I
J

## Slide 2
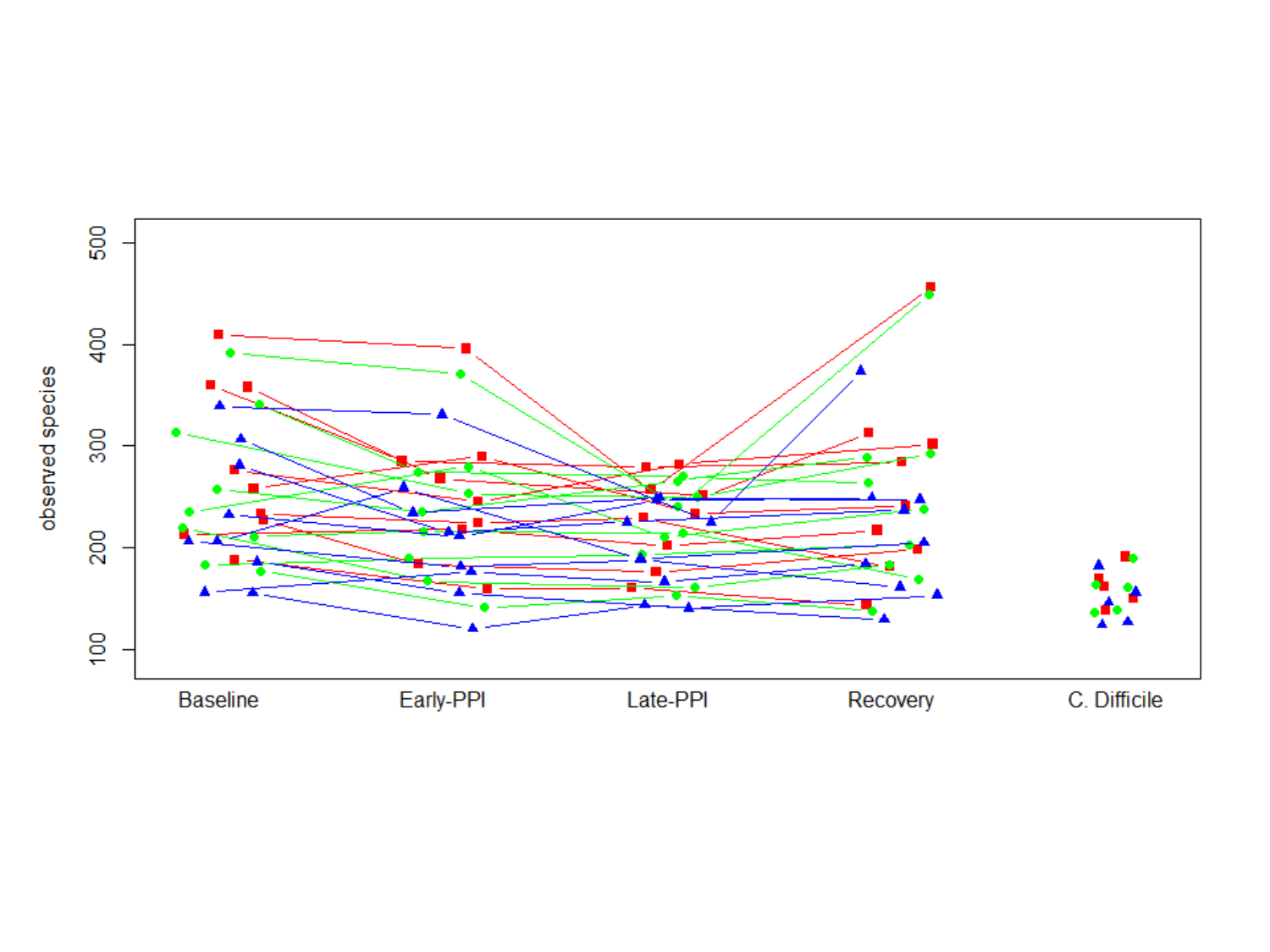

#

## Slide 3
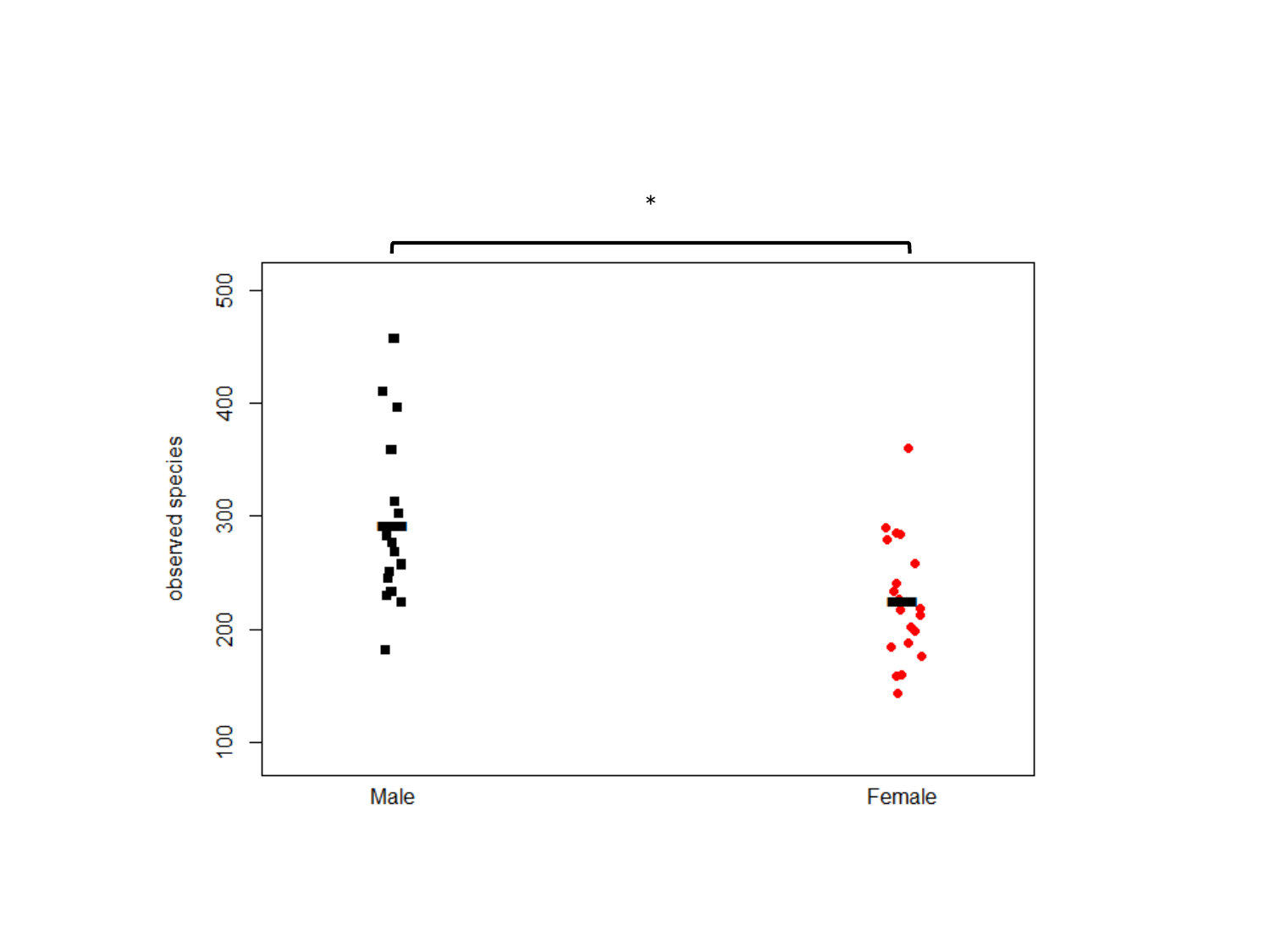

#
*

## Slide 4
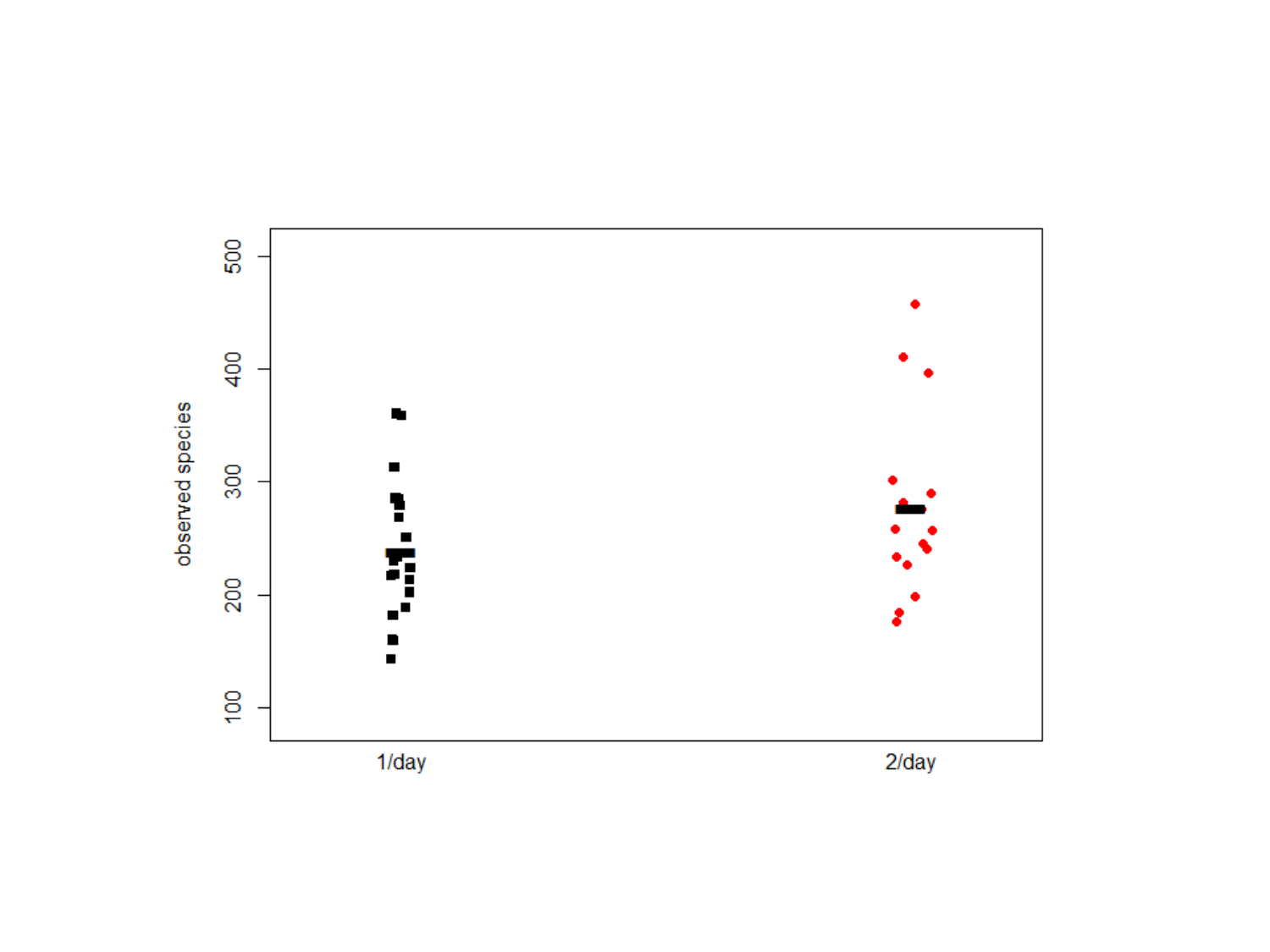

#

## Slide 5
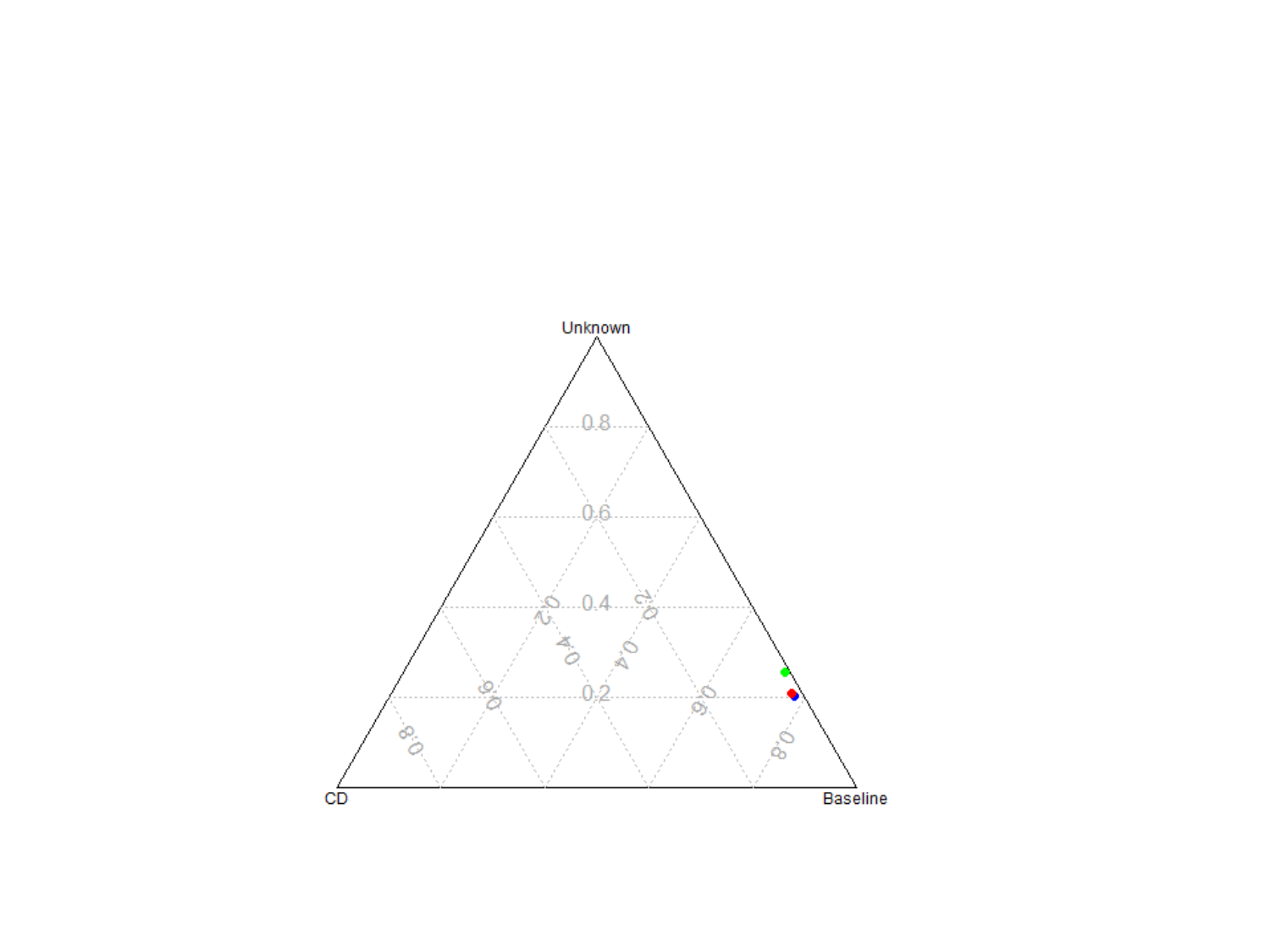

#

## Slide 6
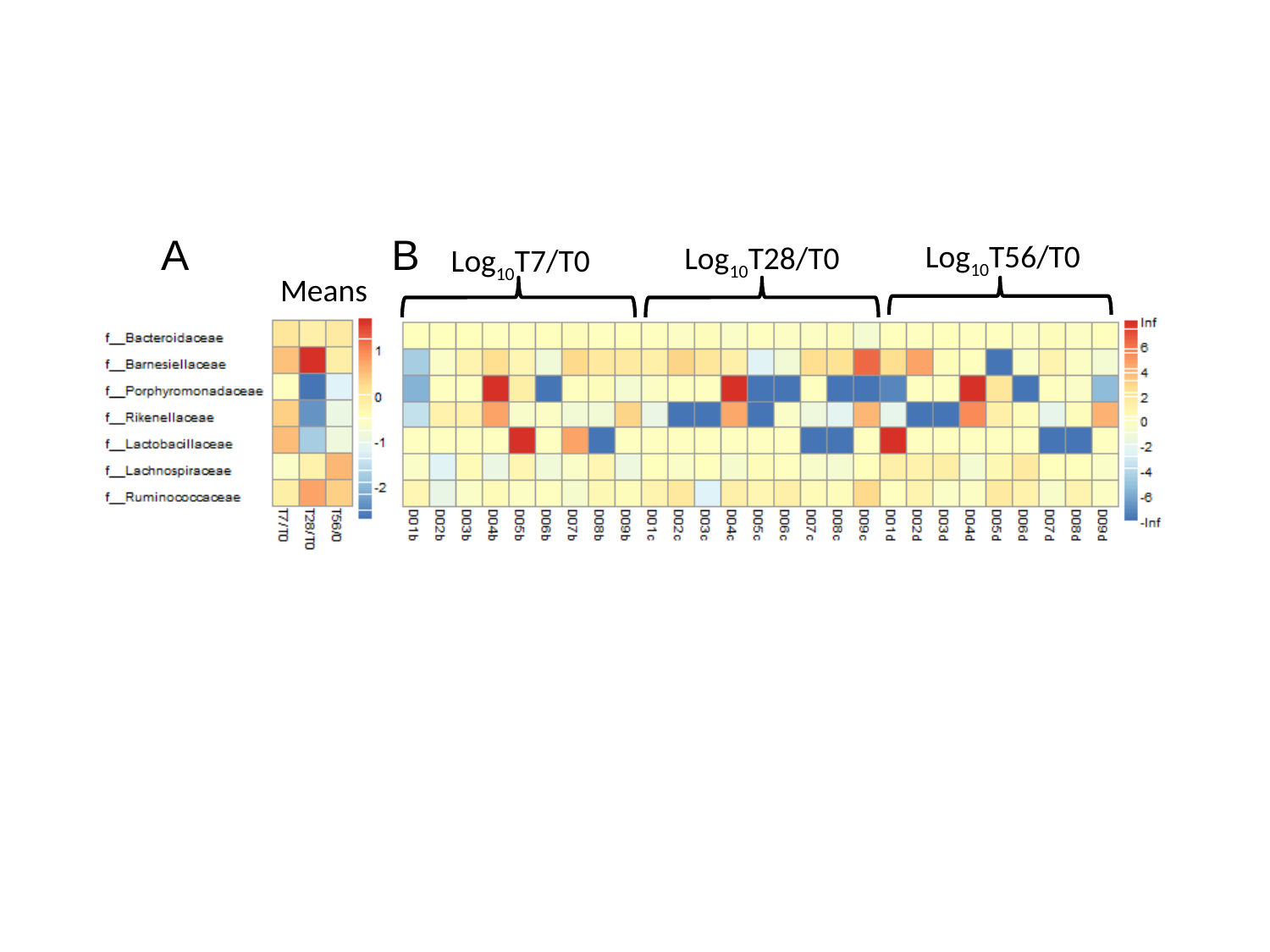

#
A
B
Log10T56/T0
Log10T28/T0
Log10T7/T0
Means

## Slide 7
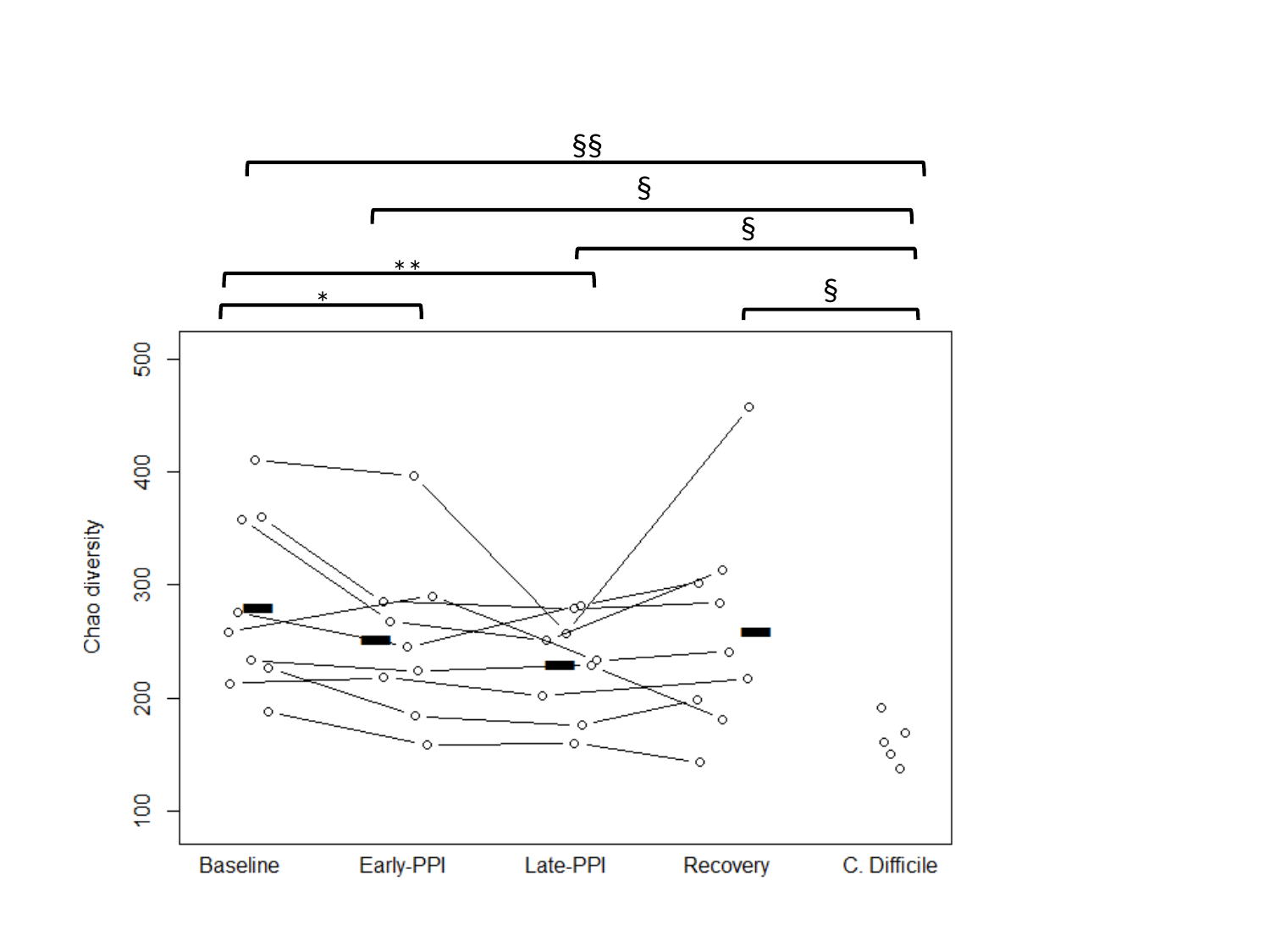

#
§§
§
§
**
§
*

## Slide 8
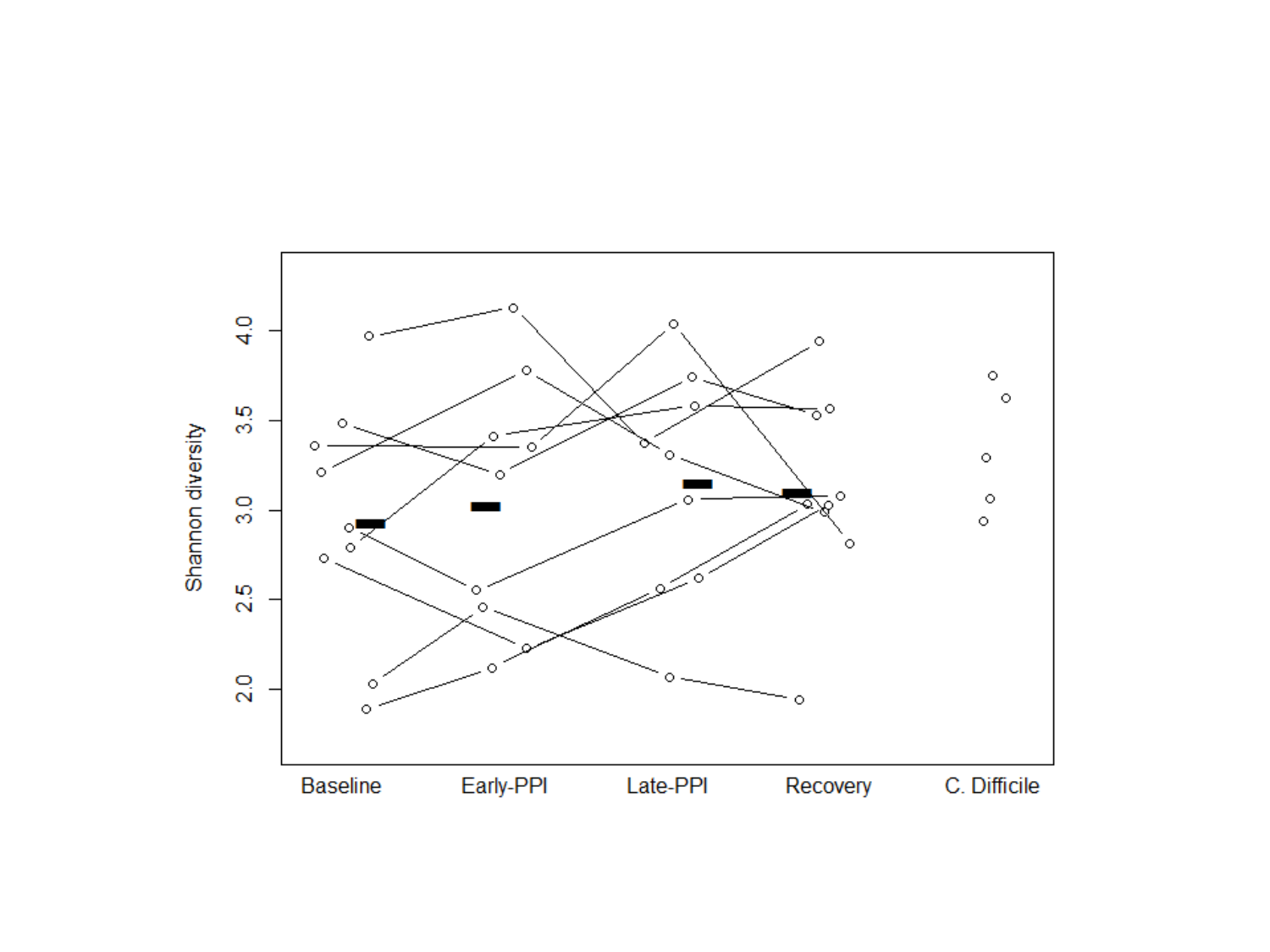

#

## Slide 9
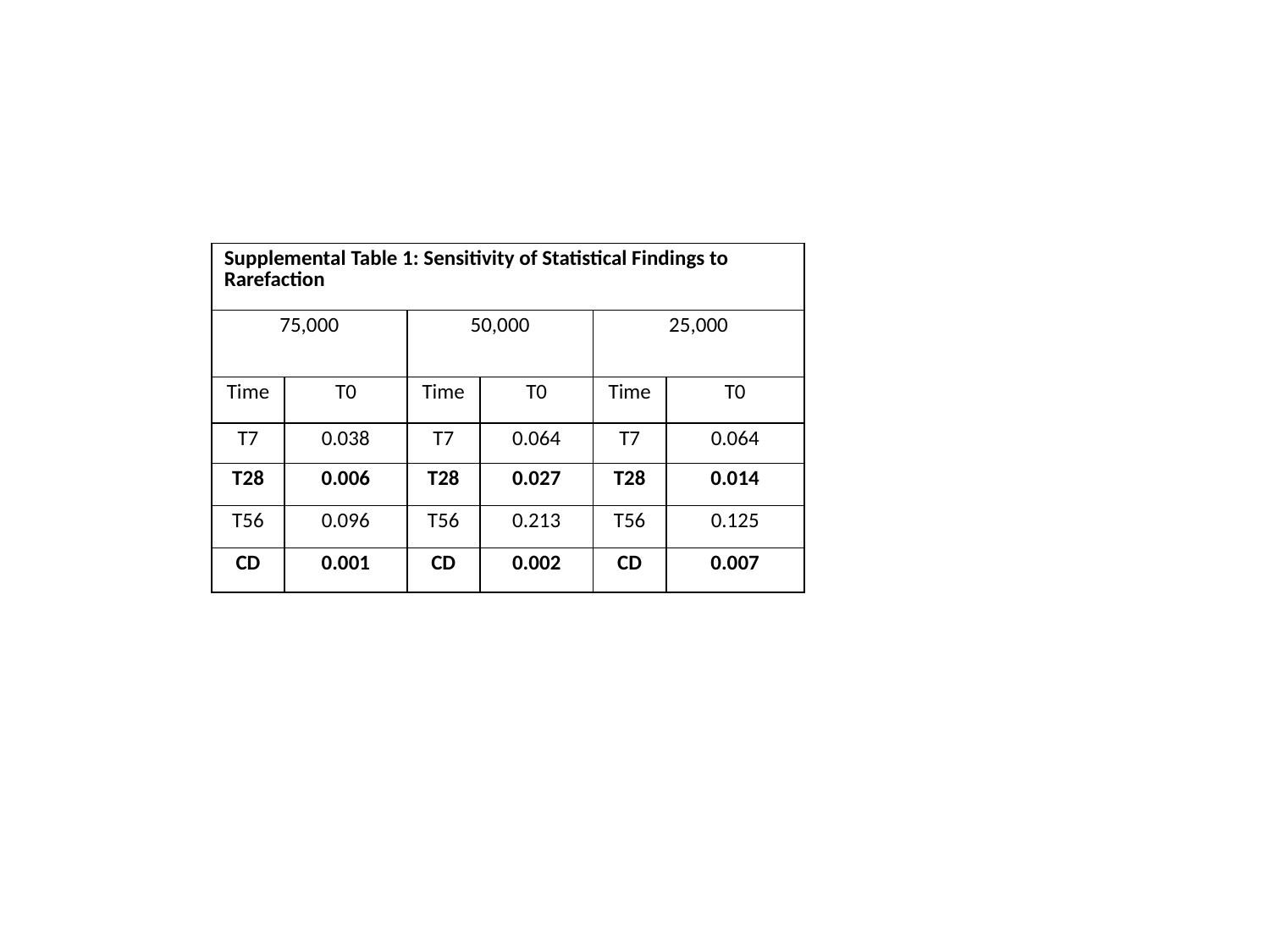

#
| Supplemental Table 1: Sensitivity of Statistical Findings to Rarefaction | | | | | |
| --- | --- | --- | --- | --- | --- |
| 75,000 | | 50,000 | | 25,000 | |
| Time | T0 | Time | T0 | Time | T0 |
| T7 | 0.038 | T7 | 0.064 | T7 | 0.064 |
| T28 | 0.006 | T28 | 0.027 | T28 | 0.014 |
| T56 | 0.096 | T56 | 0.213 | T56 | 0.125 |
| CD | 0.001 | CD | 0.002 | CD | 0.007 |

## Slide 10
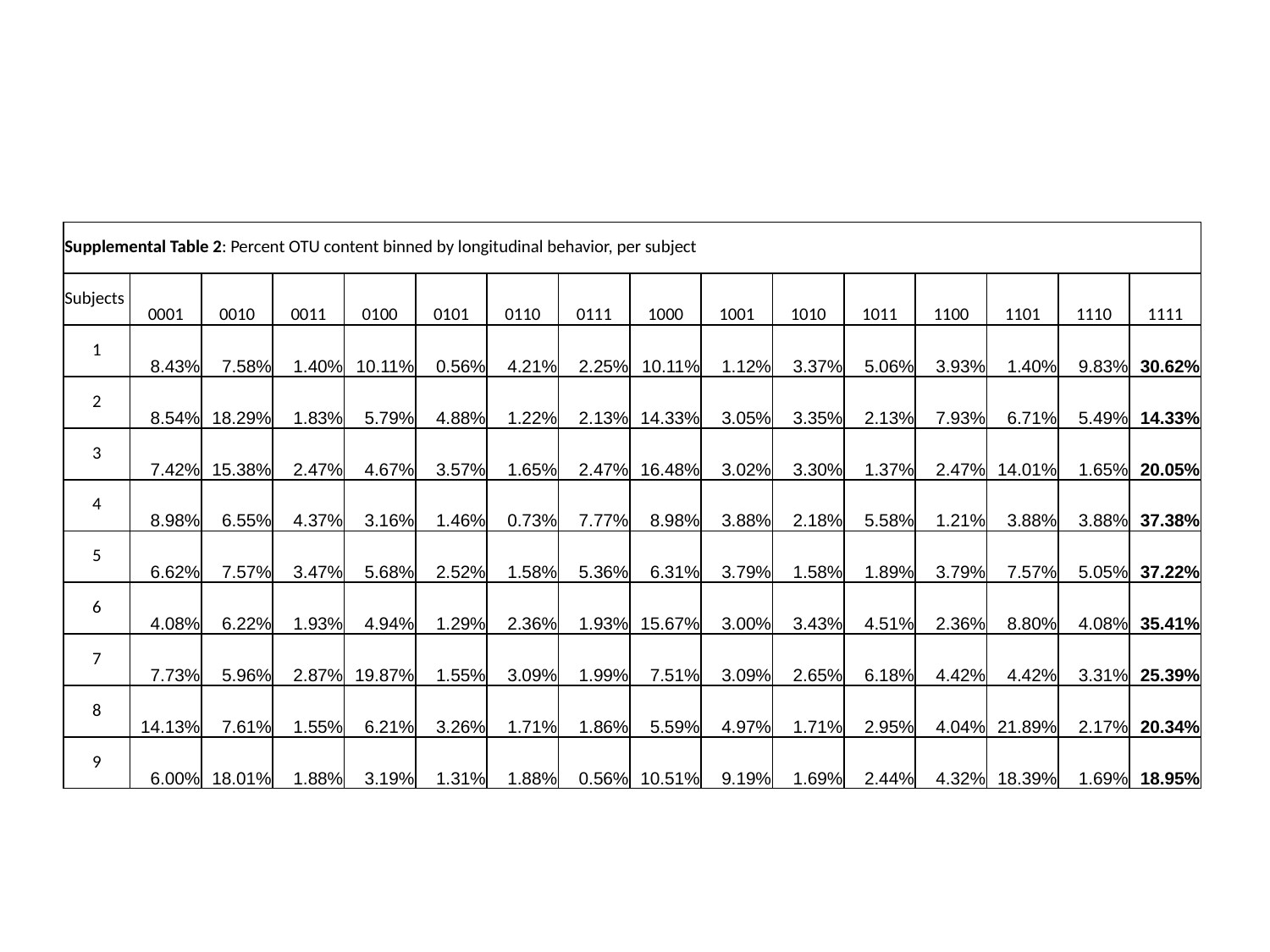

#
| Supplemental Table 2: Percent OTU content binned by longitudinal behavior, per subject | | | | | | | | | | | | | | | |
| --- | --- | --- | --- | --- | --- | --- | --- | --- | --- | --- | --- | --- | --- | --- | --- |
| Subjects | 0001 | 0010 | 0011 | 0100 | 0101 | 0110 | 0111 | 1000 | 1001 | 1010 | 1011 | 1100 | 1101 | 1110 | 1111 |
| 1 | 8.43% | 7.58% | 1.40% | 10.11% | 0.56% | 4.21% | 2.25% | 10.11% | 1.12% | 3.37% | 5.06% | 3.93% | 1.40% | 9.83% | 30.62% |
| 2 | 8.54% | 18.29% | 1.83% | 5.79% | 4.88% | 1.22% | 2.13% | 14.33% | 3.05% | 3.35% | 2.13% | 7.93% | 6.71% | 5.49% | 14.33% |
| 3 | 7.42% | 15.38% | 2.47% | 4.67% | 3.57% | 1.65% | 2.47% | 16.48% | 3.02% | 3.30% | 1.37% | 2.47% | 14.01% | 1.65% | 20.05% |
| 4 | 8.98% | 6.55% | 4.37% | 3.16% | 1.46% | 0.73% | 7.77% | 8.98% | 3.88% | 2.18% | 5.58% | 1.21% | 3.88% | 3.88% | 37.38% |
| 5 | 6.62% | 7.57% | 3.47% | 5.68% | 2.52% | 1.58% | 5.36% | 6.31% | 3.79% | 1.58% | 1.89% | 3.79% | 7.57% | 5.05% | 37.22% |
| 6 | 4.08% | 6.22% | 1.93% | 4.94% | 1.29% | 2.36% | 1.93% | 15.67% | 3.00% | 3.43% | 4.51% | 2.36% | 8.80% | 4.08% | 35.41% |
| 7 | 7.73% | 5.96% | 2.87% | 19.87% | 1.55% | 3.09% | 1.99% | 7.51% | 3.09% | 2.65% | 6.18% | 4.42% | 4.42% | 3.31% | 25.39% |
| 8 | 14.13% | 7.61% | 1.55% | 6.21% | 3.26% | 1.71% | 1.86% | 5.59% | 4.97% | 1.71% | 2.95% | 4.04% | 21.89% | 2.17% | 20.34% |
| 9 | 6.00% | 18.01% | 1.88% | 3.19% | 1.31% | 1.88% | 0.56% | 10.51% | 9.19% | 1.69% | 2.44% | 4.32% | 18.39% | 1.69% | 18.95% |

## Slide 11
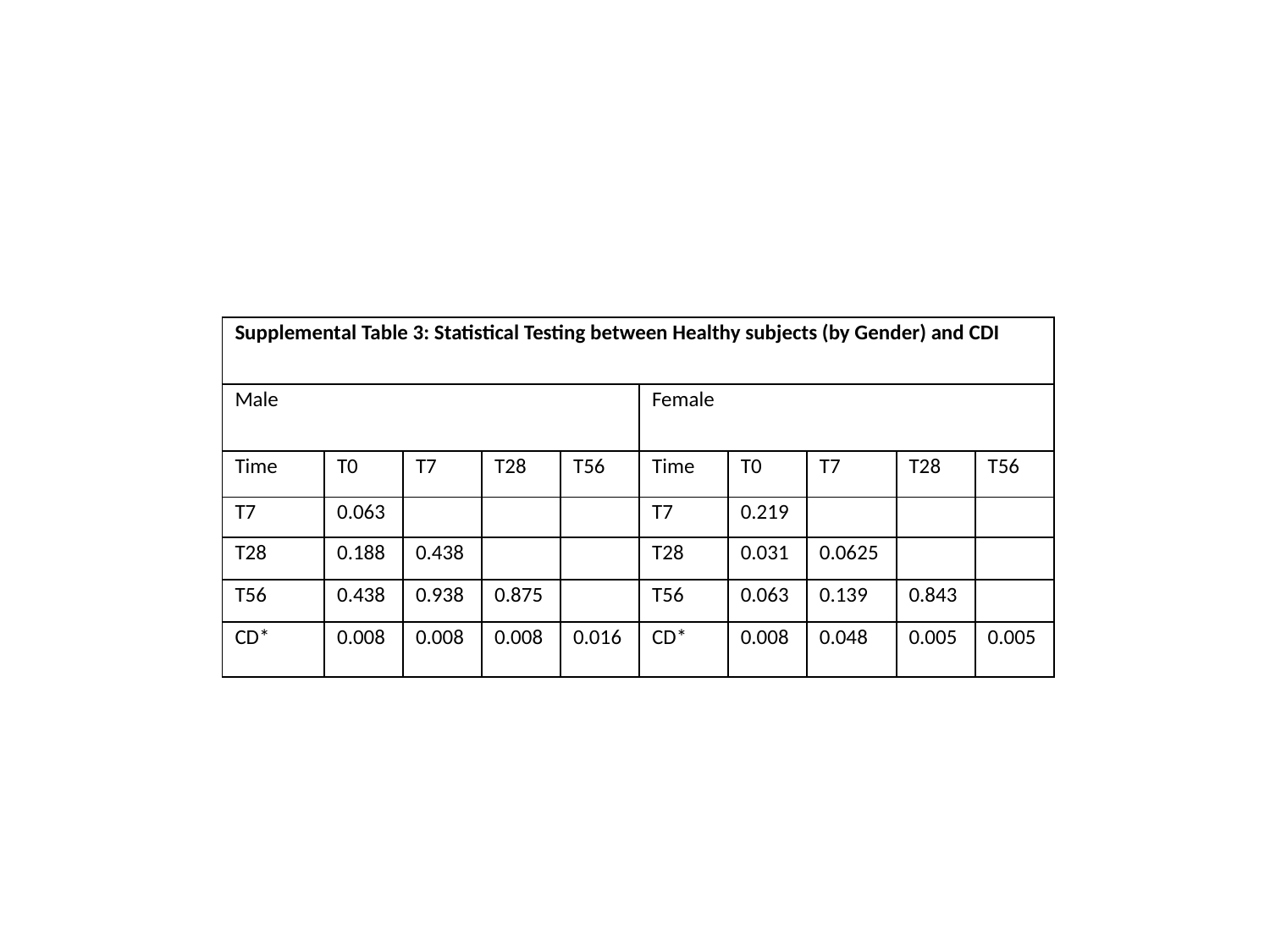

#
| Supplemental Table 3: Statistical Testing between Healthy subjects (by Gender) and CDI | | | | | | | | | |
| --- | --- | --- | --- | --- | --- | --- | --- | --- | --- |
| Male | | | | | Female | | | | |
| Time | T0 | T7 | T28 | T56 | Time | T0 | T7 | T28 | T56 |
| T7 | 0.063 | | | | T7 | 0.219 | | | |
| T28 | 0.188 | 0.438 | | | T28 | 0.031 | 0.0625 | | |
| T56 | 0.438 | 0.938 | 0.875 | | T56 | 0.063 | 0.139 | 0.843 | |
| CD\* | 0.008 | 0.008 | 0.008 | 0.016 | CD\* | 0.008 | 0.048 | 0.005 | 0.005 |

## Slide 12
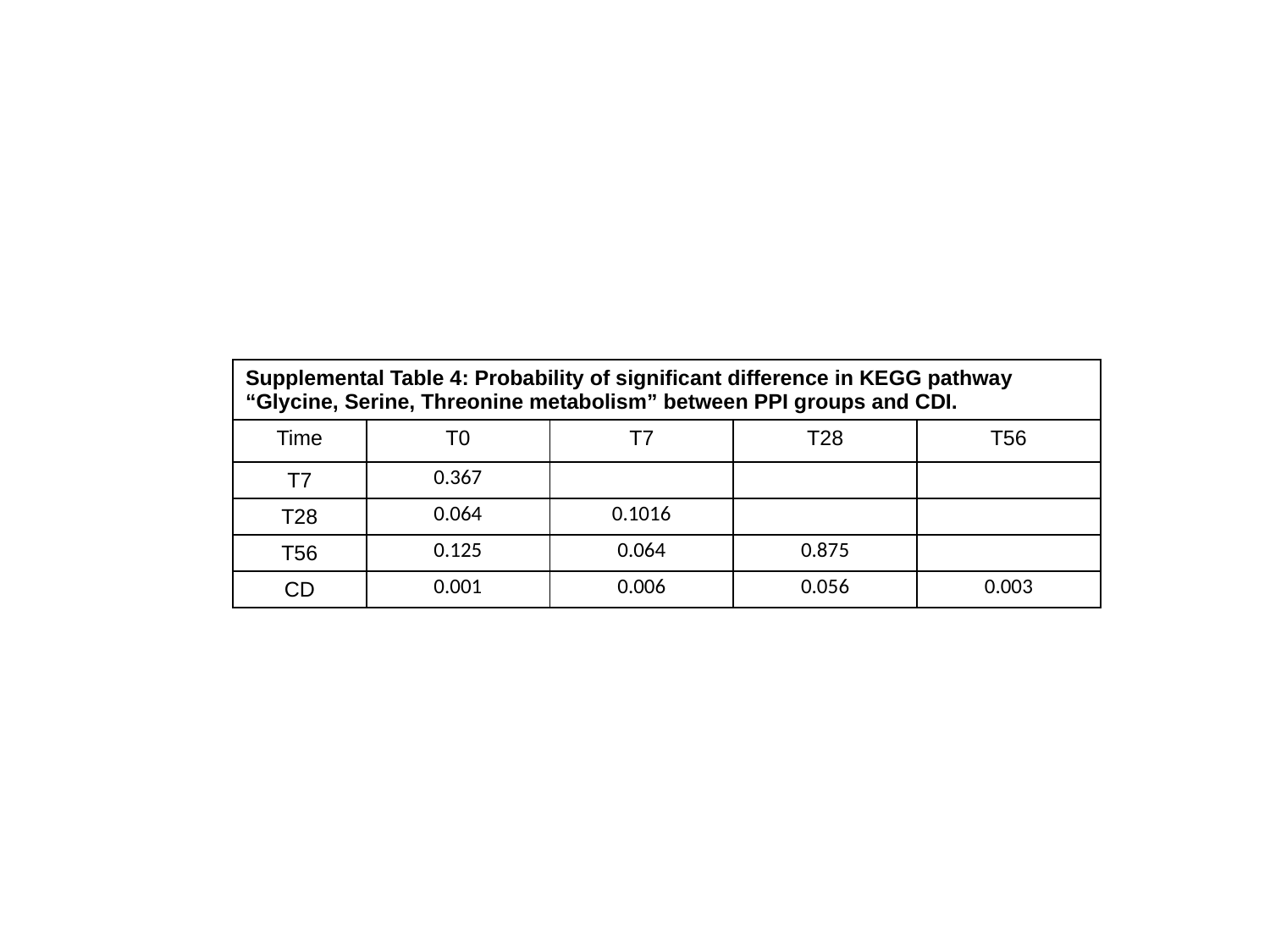

#
| Supplemental Table 4: Probability of significant difference in KEGG pathway “Glycine, Serine, Threonine metabolism” between PPI groups and CDI. | | | | |
| --- | --- | --- | --- | --- |
| Time | T0 | T7 | T28 | T56 |
| T7 | 0.367 | | | |
| T28 | 0.064 | 0.1016 | | |
| T56 | 0.125 | 0.064 | 0.875 | |
| CD | 0.001 | 0.006 | 0.056 | 0.003 |

## Slide 13
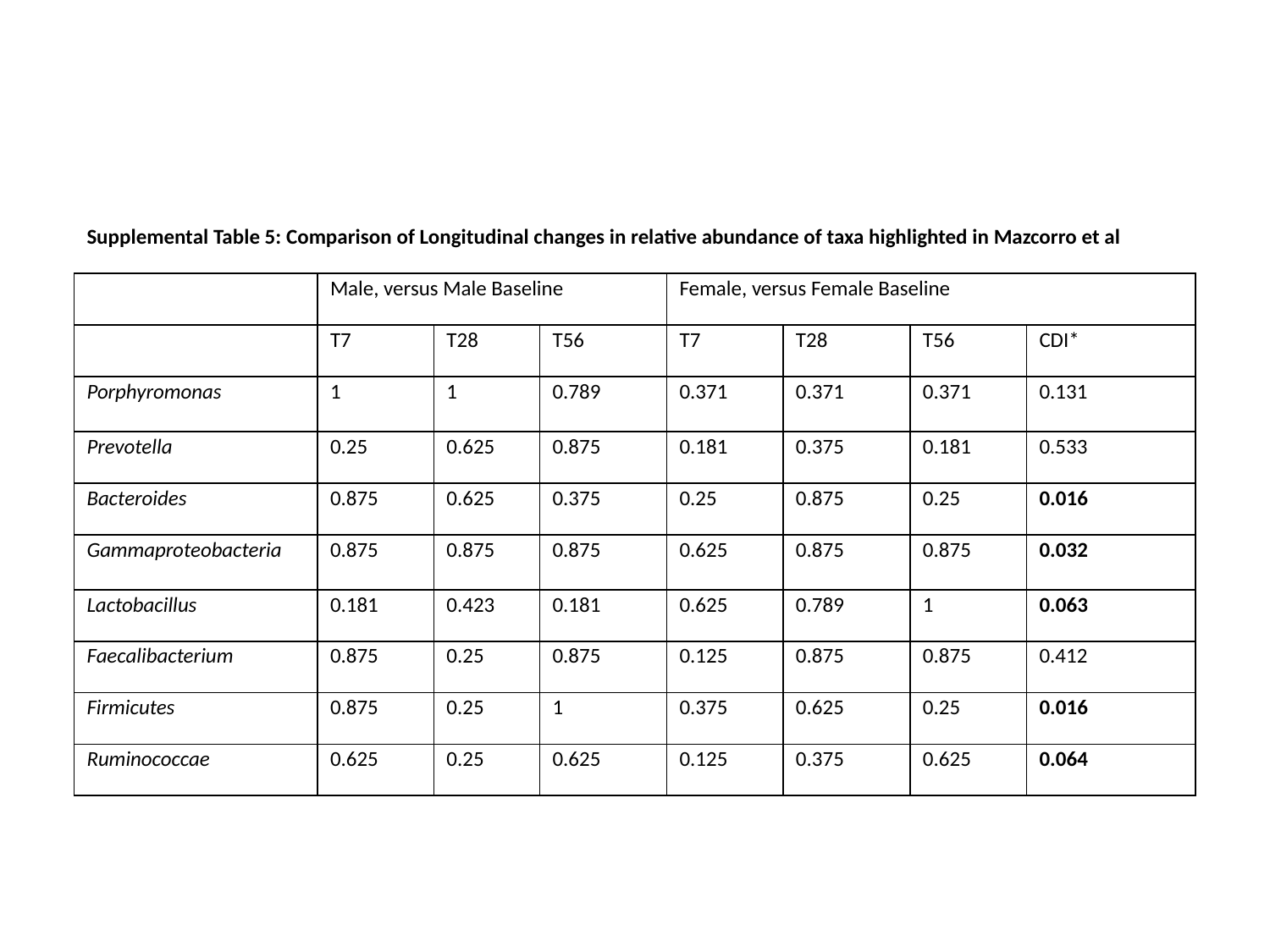

#
| Supplemental Table 5: Comparison of Longitudinal changes in relative abundance of taxa highlighted in Mazcorro et al | | | | | | | |
| --- | --- | --- | --- | --- | --- | --- | --- |
| | Male, versus Male Baseline | | | Female, versus Female Baseline | | | |
| | T7 | T28 | T56 | T7 | T28 | T56 | CDI\* |
| Porphyromonas | 1 | 1 | 0.789 | 0.371 | 0.371 | 0.371 | 0.131 |
| Prevotella | 0.25 | 0.625 | 0.875 | 0.181 | 0.375 | 0.181 | 0.533 |
| Bacteroides | 0.875 | 0.625 | 0.375 | 0.25 | 0.875 | 0.25 | 0.016 |
| Gammaproteobacteria | 0.875 | 0.875 | 0.875 | 0.625 | 0.875 | 0.875 | 0.032 |
| Lactobacillus | 0.181 | 0.423 | 0.181 | 0.625 | 0.789 | 1 | 0.063 |
| Faecalibacterium | 0.875 | 0.25 | 0.875 | 0.125 | 0.875 | 0.875 | 0.412 |
| Firmicutes | 0.875 | 0.25 | 1 | 0.375 | 0.625 | 0.25 | 0.016 |
| Ruminococcae | 0.625 | 0.25 | 0.625 | 0.125 | 0.375 | 0.625 | 0.064 |

## Slide 14
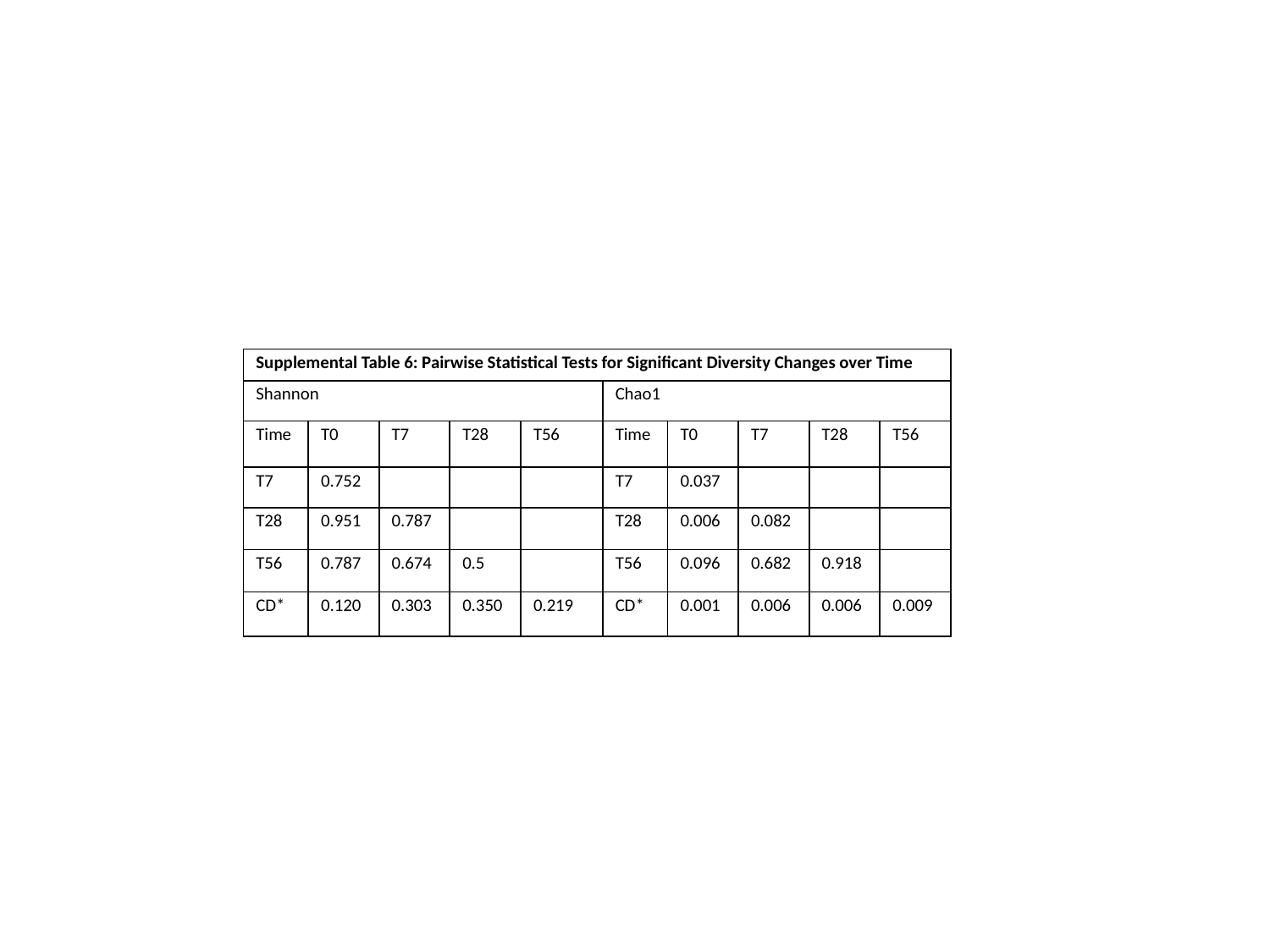

#
| Supplemental Table 6: Pairwise Statistical Tests for Significant Diversity Changes over Time | | | | | | | | | |
| --- | --- | --- | --- | --- | --- | --- | --- | --- | --- |
| Shannon | | | | | Chao1 | | | | |
| Time | T0 | T7 | T28 | T56 | Time | T0 | T7 | T28 | T56 |
| T7 | 0.752 | | | | T7 | 0.037 | | | |
| T28 | 0.951 | 0.787 | | | T28 | 0.006 | 0.082 | | |
| T56 | 0.787 | 0.674 | 0.5 | | T56 | 0.096 | 0.682 | 0.918 | |
| CD\* | 0.120 | 0.303 | 0.350 | 0.219 | CD\* | 0.001 | 0.006 | 0.006 | 0.009 |
